# 3D ultra-short echo time ^31^P-MRSI with rosette k-space pattern: Feasibility and comparison with conventional weighted CSI

**DOI:** 10.21203/rs.3.rs-4223790/v1

**Published:** 2024-04-08

**Authors:** Brian Bozymski, Uzay Emir, Ulrike Dydak, Xin Shen, M. Albert Thomas, Ali Özen, Mark Chiew, William Clarke, Stephen Sawiak

**Affiliations:** School of Health Sciences, Purdue University; School of Health Sciences, Purdue University; School of Health Sciences, Purdue University; Radiology and Biomedical Imaging, University of California San Francisco; Department of Radiology, University of California; Department of Radiology, Medical Physics, Medical Center - University of Freiburg; Wellcome Centre for Integrative Neuroimaging, FMRIB, Nuffield Department of Clinical Neurosciences; Wellcome Centre for Integrative Neuroimaging, FMRIB, Nuffield Department of Clinical Neurosciences; Department of Clinical Neuroscience, University of Cambridge

## Abstract

Phosphorus-31 magnetic resonance spectroscopic imaging (^31^P-MRSI) provides valuable non-invasive *in vivo* information on tissue metabolism but is burdened by poor sensitivity and prolonged scan duration. Ultra-short echo time (UTE) acquisitions minimize signal loss when probing signals with relatively short spin-spin relaxation time (T_2_), while also preventing first-order dephasing. Here, a three-dimensional (3D) UTE sequence with a rosette k-space trajectory is applied to ^31^P-MRSI at 3T. Conventional chemical shift imaging (CSI) employs highly regular Cartesian k-space sampling, susceptible to substantial artifacts when accelerated via undersampling. In contrast, this novel sequence’s “petal-like” pattern offers incoherent sampling more suitable for compressed sensing (CS). These results showcase the competitive performance of UTE rosette ^31^P-MRSI against conventional weighted CSI with simulation, phantom, and *in vivo* leg muscle comparisons.

## 1. Introduction

Phosphorous-31 magnetic resonance spectroscopy (^31^P-MRS), the longest-standing *in vivo* MRS modality, can be an invaluable tool for probing *in vivo* metabolites such as phosphocreatine (PCr), inorganic phosphate (Pi), phosphomonoesters (PMEs), phosphodiesters (PDEs), and adenosine triphosphate (ATP) .^1,2^ As fundamental phospholipids and consitituents of the high-energy phosphate pathway, these ^31^P metabolites provide noninvasive measures of tissue pH, lipid metabolism, and oxidative bioenergetics.^3,4^ Thus, ^31^P-MRS possesses versatile diagnostic and prognostic potential. For instance, elevated PME/PDE ratios and reduced ATP levels have been reported in diseased and cancerous liver tissue, often correlated with classical plasma markers and Child-Pugh scores.^5–9^ Furthermore, ^31^P-MRS has been used to assess whole-liver treatment efficacy, monitoring metabolite changes in malignant tissues following therapy.^10^ Likewise, diminished PCr/ATP ratios and post-exercise PCr recovery rates have been measured in cardiac and skeletal muscles of patients with type 2 diabetes.^11^ Numerous endeavors have employed ^31^P MRS in the brain, heart, and muscle, seeking out alterations in neurodegenerative, cardiovascular, metabolic, and oncological diseases.^12–20^

While relevant ^1^H-MRS metabolite signals are obscured by background signals such as contaminating fat, water, and macromolecular signals, widely spaced ^31^P spectral peaks can be easily elucidated due to the absence of these nuisance signals. However, in contrast to ^1^H-MRS, ^31^P-MRS is burdened by a lower gyromagnetic ratio and relatively short spin-spin metabolite relaxation times (T_2_);^21,22^ these factors engender extremely poor *in vivo* relative sensitivity and force a delicate balance between SNR, resolution, and scan duration. Low ^31^P-MRS tissue concentrations (approximately 2 mM γ-ATP in liver^23^) further exacerbate SNR challenges, so that commonly used acquisition delays (T_E_ > 300 μs) with conventional methods result in prolonged acquisition, phase distortions, baseline roll, and subsequent operator errors during metabolite quantification. Such complications have been severely limiting factors in the clinical feasibility of ^31^P-MRS. Recent advances in coil engineering and the introduction of ultra-high field (UHF, B_0_ > 3T) scanners have assisted in mitigating these limiting factors; one experiment demonstrated a 2.8-factor increase in PCr SNR at 7T relative to 3T.^24^ Conversely, UHF acquisitions also necessitate larger spectral bandwidth (SBW), with a 40 ppm range requiring approximately 2.0 kHz at 3T but 4.8 kHz at 7T. Still, excessive acquisition durations remain the clear barrier to clinical translation without innovative acceleration.

**Figure 1:**
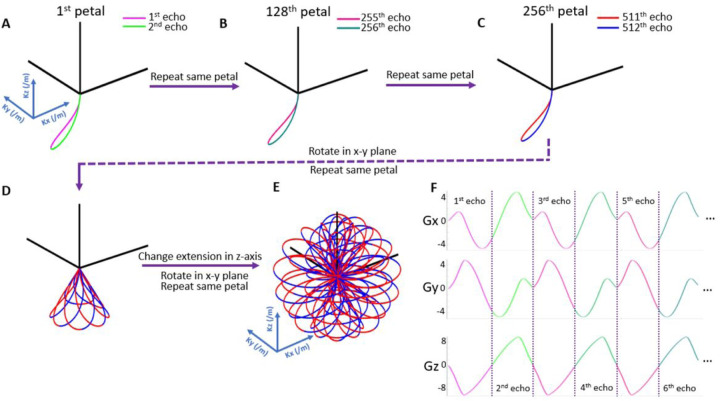
Illustration of 3D rosette k-space trajectory and gradients. **(A-C)** Acquisition begins at k-space center for every petal, crossing k-space origin twice at each petal’s beginning and end. Petals can be manually separated into two halves, similar to odd and even echoes in EPSI MRSI. **(D, E)** Varied petal rotations form the rosette pattern, providing sufficient k-space coverage. **(F)** With the closed-loop trajectory, acquisition delay is further minimized by enabling the analog to digital converter (ADC) for sampling during gradient ramp-up.

To address these points, we propose a three-dimensional (3D), ultra-short echo time (UTE) sequence with a novel rosette k-space trajectory (previously validated in ultra-short-T_2_ imaging^25,26^ and brain iron mapping^27,28^) for ^31^P magnetic resonance spectroscopic imaging (MRSI).^29^ Compared to conventional CSI Cartesian k-space trajectories, rosette’s “petal-like” pattern (Fig. 1) maps 3D k-space far more efficiently. Additionally, rosette’s relatively inchorent data sampling allows the possibility of significant acceleration through higher undersampling factors and compressed sensing (CS) reconstruction; offering better k-space coverage when compared to radial and spiral trajectories, generalized rosette’s curvature affords superior SNR performance under aggressive acceleration.^30^ Furthermore, UTE acquisitions permit the capture of short-T_2_ signals before significant transverse signal decay and first-order dephasing occur, enhancing SNR, simplifying spectral pre-processing, and minimizing operator quantification errors.

Substantial efforts have been invested towards clinically feasible ^31^P-MRSI, experimenting with short repetition times (T_R_), measuring multiple k-space points per T_R_, k-space undersampling, enhanced reconstruction via prior knowledge, and their conceivable combinations.^31^ 3D extensions of ISIS have shown promise in UHF preclinical and 3T cardiac studies but remain limited by time resolution and motion artifact sensitivity.^32,33^ Non-localized or FID acquisitions are often preferred to minimize rapid ^31^P T_2_-decay, but also to overcome specific absorption rate (SAR) limitations at UHFs. Thus, variations of spatial-spectral encoding (SSE) schemes and their synergies with k-space undersampling appear to be the more promising avenue forward; several Cartesian and non-Cartesian acquisition designs offer varying degrees of SNR efficiency, k-space weighting, gradient system demands, and undersampling acceleration potential.

Flyback EPSI has been tested in skeletal calf muscle,^34^ offering considerable time savings over conventional phase-encoded when combined with CS acceleration;^35^ despite its acceleration potential, EPSI offers lower SNR efficiency and SBW at fine resolutions than other SSE options. Density-weighted concentric ring^36^ trajectories (CRTs) offer increased SNR efficiency and SBW limits, enabling faster MRSI even with UHF systems. CRTs boast flexibility in weighting and temporal interleaves, allowing tailoring according to acceleration needs and gradient slew rates. Similarly, spiral-encoded ^31^P-MRSI has exhibited faster dynamic calf muscle mapping than conventional weighting phase-encoded acquisition.^37^ Though spirals offer high acceleration, SNR efficiency, and customizable weighting, they are also limited by SBW and gradient system hardware.

Conventional CSI FIDs commonly possess T_E_ on the order of 1–2 ms, which is restricted by the duration of constant excitation pulses and phase encoding gradients to reach the outermost k-space points. This acquisition delay can be decreased using variable pulse widths and amplitudes. Sampling throughout gradient transition periods, or ramp sampling, remains an additional option for minimizing T_E_. Studies have showcased T_E_ as low as 480 μs and 520 μs for EPSI and radial EPSI, respectively.^35,38^ Acquisitions using such UTE CSI techniques^39^ have achieved T_E_ = 300 μs at 3T and T_E_ = 500 μs at 7T.^40,41^ In non-Cartesian center-out k-space trajectories employing ramp sampling, T_E_ is primarily limited by the dead time between coil transmit-receive switching, permitting the shortest possible T_E_. Despite this possibility, SBW constraints and conventional sequence parameter (T_A_/FOV) matching have prevented extensive investigation of non-Cartesian UTE ^31^P-MRSI.

In this study, we evaluate the 3D UTE ^31^P-MRSI with a novel rosette k-space trajectory by comparing its performance to conventional 3D-weighted ^31^P CSI in the quadriceps muscle at 3T. We ultimately aim to demonstrate its potential value in clinical spectroscopic acquisitions.

## 2. Methods

### 2.1 k-space trajectory designs for MRSI

#### 2.1.1 UTE Rosette

General sequence parameters were as follows: T_A_ = 36:00, T_R_ = 350 ms, T_E_ = 65 μs, matrix size = 24×24×24, nominal voxel size = 8 mL, FOV = (480×480×480) mm^3^, SBW = 2083 Hz, spectral time samples = 512. Parameters are summarized in Table 1.

As in prior work,^25^ 3D UTE rosette k-space trajectory (Fig. 1) for ^31^P-MRSI was generated with Equations (1) and (2):

$$K_{\mathrm{xy}}=kx(t)+i*Ky(t)$$

(1) $$=(K\text{max}\;*\;\text{cos}\;(\phi ))\;*\;\text{sin}\;(\omega_{\text{1}}\;*\;t)\;*\;{\text{e}}^{i\omega_{\text{2}}t+\beta }$$

(2) $$K_{z}(t)=(K\text{max}\;*\;\sin\;(\phi ))\;*\;\text{sin}\;(\omega_{\text{1}}\;*\;t)$$

Where *K* max is the maximum extent of k-space, *ω*1 is the frequency of oscillation in the radial direction, *ω*2 is the frequency of rotation in the angular direction, *ϕ* determines the location in the z-axis, and *β* determines the initial phase in the angular direction. For this ^31^P-MRSI study, *K* max = 25/m, *ϕ* was sampled uniformly in the range of [-π/2, π/2], was sampled uniformly in the range of [0, 2π], RF pulse duration = 50 μs, readout dwell time = 5 μs, and each rosette petal was designed with 96 points. This leads to

$$\omega_{1}=\omega_{2}=\frac{\pi }{\text{pointsperpetal}(N_{pp})*\text{dwelltime}}=\frac{\pi }{96*5\mu \text{s}}=6545\text{rad}/\text{s}$$

as well as

$$\text{SBW}=\frac{1}{N_{pp}*\text{dwelltime}}=\frac{\pi }{96*5\mu \text{s}}={\left(480\mu \text{s}\right)}^{-1}=2083\text{Hz}$$

and the resulting − 20 to + 20 ppm 3T spectral range is more than sufficient for ^31^P-MRS. In reconstruction, each petal was downsampled from *N*_*pp*_ = 96 points to *N*_*pp*_ = 48 by averaging the oversampled points. With a 24×24×24 reconstruction matrix, the required number of petals (*N*_*p*_) to satisfy Nyquist criterion was calculated as

$$N_{p}=4\pi *{\left(\frac{24}{2}\right)}^{2}=1810$$

However, due to the rosette’s efficient sampling scheme, only 80% coverage (*N*_*p*_ = 1444) was defined as full k-space acquisition. Thus, the acquisition time per average was calculated as

$${\text{T}}_{A}=N_{p}*\;\;{\text{T}}_{R}=1444\;*\;350\text{ms}=505\text{s}$$

or roughly 9 minutes. A complete description including the influence of trajectory parameters, Nyquist criterion, and the specific gradient ramp-up of this 3D rosette k-space pattern is provided in earlier work (Shen et al.).^25^

#### 2.1.2 Weighted CSI

Conventional Cartesian 3D acquisitions used the vendor-provided ^31^P CSI FID with k-space weighting and Hanning filter. Sequence parameters were as follows: T_A_ = 36:56, T_R_ = 1000 ms, T_E_ = 2.3 ms, matrix size = 16×16×16, nominal voxel size = 8 mL, FOV = (320×320×320) mm^3^, SBW = 2200 Hz, spectral time samples = 512. Parameters are summarized in Table 1.

### 2.2 Simulations

To assess the theoretical performance of the 3D UTE rosette relative to weighted CSI, MATLAB (Mathworks, Natick, USA) simulations were run examining side lobes and SNR relative to the spatial response function (SRF). A simple, constant 3D object was placed at the origin of a 48 × 48 × 48 grid (FOV = 480 mm isotropic producing a 1 mL nominal voxel size) with added noise and reconstructed using the non-uniform FFT (NUFFT) method and k-space information for each *in vivo* acquisition. We use spatial response function (SRF) instead of point spread function (PSF) since the former specifically estimates side lobes and signal bleed between adjacent voxels, while the latter measures contribution from a single object point to the entire population of voxels.

### 2.3 Experimental comparison

All data acquisition occurred on a 3T MRI system (Prisma, Siemens, Erlangen, Germany) with G_max_ = 80 mT/m and slew rate = 200 mT/m/ms isotropically. Human subject protocols were approved by the Institutional Review Boards of Purdue University, and informed consent was obtained. Sequence parameters are summarized in Table 1.

The rosette and conventional acquisitions were tested with a uniform 2-liter bottle phantom (0.17 mg/mL phosphoric acid) using a dual-tuned ^1^H/^31^P Tx/Rx flexible 11-cm surface coil (RAPID Biomedical). For *in vivo* comparison, five healthy volunteers (BMI = 26 ± 2 kg/m^2^; age = 29 ± 5 years; 2 f / 3 m) underwent leg scans with an 8-channel, dual-tuned ^1^H/^31^P Tx/Rx phased array coil^42^ (Stark Contrast, Erlangen,Germany). Quadriceps was chosen for its superior PCr SNR and absence of respiratory motion during prolonged scanning. Subjects were positioned feet-first and supine, with the upper quadriceps tightly surrounded by the coil plates. Following localizer imaging, the adjustment volume was manually positioned (spanning both legs), and linewidth was minimized using a 3D GRE field map and interactive SIEMENS shimming. Each subject was scanned first with the conventional weighted Cartesian acquisition followed uninterrupted by the 3D UTE rosette ^31^P-MRSI. Sequence parameters are summarized in Table 1.

### 2.4 Post-processing and reconstruction

Raw data files were exported for reconstruction and pre-processing in MATLAB. Gridding and FFT were completed using adjoint NUFFT (regridding),^43^ Hanning filtered, and, when necessary, coil-combined using whitened singular value decomposition (wSVD).^44^ Spectra were zero-order phased by maximizing the integral of the largest peak (PCr, 0 ppm) for the 3D UTE rosette. Spectra from the weighted CSI were zero-order phased and first-order phased to correct a 2.3 ms delay.

### 2.5 SNR and quantification

Spectra were fitted within the Oxford Spectroscopy Analysis (OXSA) toolbox^45^ using AMARES methods. Metabolite peak SNRs were calculated according to Eq. (3), with noise variance calculated from a residual region lacking metabolite signals. As an additional signal quantification metric, “raw SNR” (Eq. (4)) was estimated by dividing the highest absolute peak point by the noise variance in an off-spectrum region; this method carries the advantage of consistently assessing signal strength regardless of any interfering spectral phase.

(3) $${\text{SNR}}_{\text{OXSA}}=\frac{\text{PeakSignalFit}\;(\text{Real})}{{\text{RMS}}_{\text{residualnoise}}}$$

(4) $${\text{SNR}}_{\text{raw}}=\frac{\text{MaximumPeakAmplitude}\;(\text{Absolute})}{{\text{RMS}}_{\text{off-spectrumnoise}}}$$

**Figure 2:**
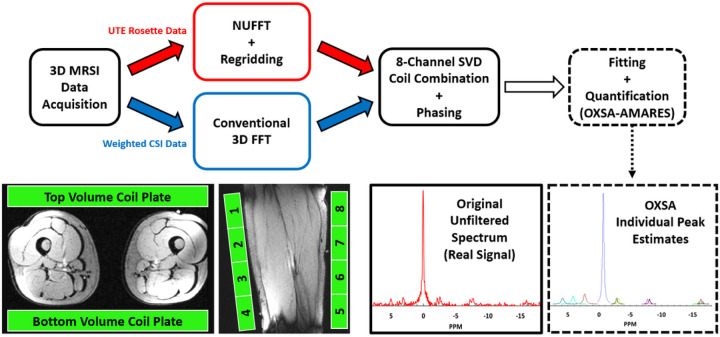
Workflow of data acquisition, reconstruction, processing, and analysis. Subjects were positioned feet-first supine with both quadriceps positioned between the 30-cm phased array coil plates. Raw data were exported, appropriately reconstructed, coil-combined, and phased prior to fitting and quantification.

**Figure 3:**
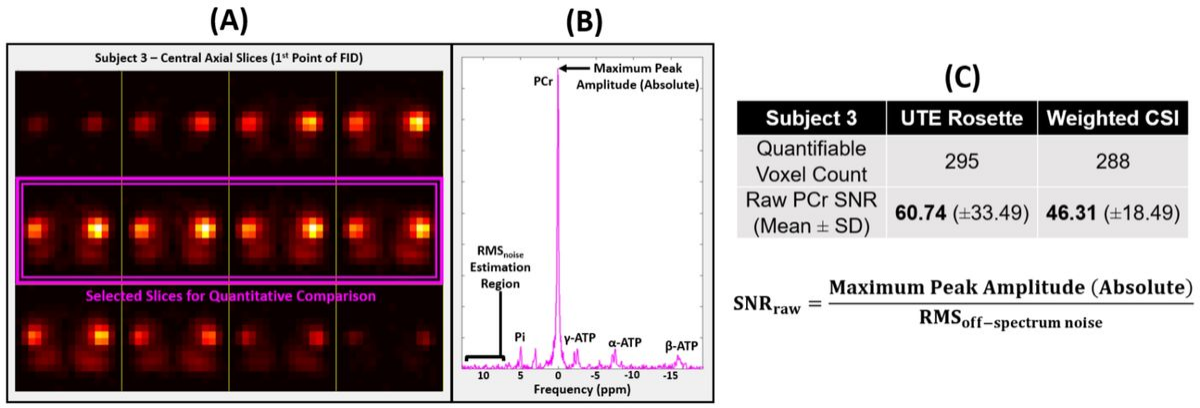
**(A)** Signal intensity and **selected central axial slices** for Subject 3’s rosette UTE acquisition. **(B)** Visualization of “raw SNR” calculation on ^31^P-MRS muscle spectrum in one voxel. **(C)** Results for quantifiable (SNR >3) voxels within **selection**.

Data acquisition, reconstruction, processing, and analysis workflow is summarized in Figs. 2 and 3.

### 2.6 Quantitative analysis

The performance of 3D UTE rosette and weighted CSI in phantom solution and quadriceps muscle were assessed using Pi and PCr metabolite signals, respectively. Quantification considered the central, highest signal axial slices within each subject, attempting to quantify every voxel. Only voxels with SNR > 3 and OXSA-AMARES Cramér-Rao lower bound (CRLB) goodness of fit smaller than 20% for PCr peak were included in the final analysis.

## 3. Results

### 3.1 Spatial Response Function simulation comparison of UTE Rosette with Weighted CSI

**Figure 4:**
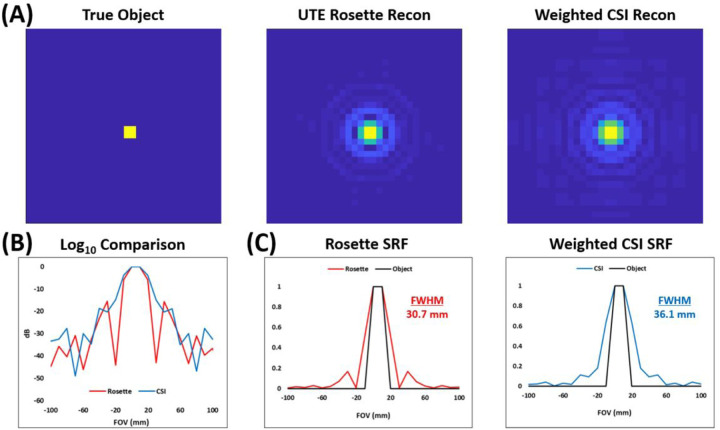
Results of spatial response function (SRF) simulation for conventional 3D weighted CSI and novel 3D rosette MRSI sequences. **(A)** 2D (xy-plane) SRFs for simulated object and each k-space trajectory at center of the FOV. **(B)** 1D (x-axis) log_10_ decibel comparison between k-space trajectories at center of the FOV. **(C)** 1D (x-axis) comparisons between each normalized reconstruction and the true simulated object at center of the FOV.

The impact of varying k-space sampling trajectories on image quality can be evaluated via SRF simulations as shown in Fig. 4. FWHMs along the x-axis at the center of the FOV were comparable between rosette (30.7 mm) and weighted CSI (36.1 mm). Both acquisition schemes exhibit noticeable sidelobe noise, albeit with slightly reduced side lobes in the rosette trajectory.

### 3.2 Phantom comparison of UTE Rosette with Weighted CSI

**Figure 5:**
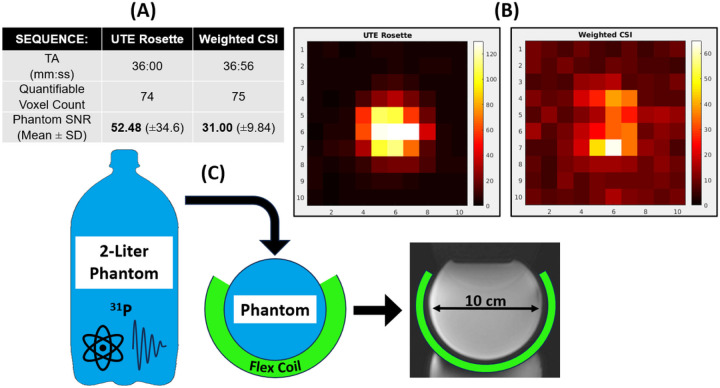
Results from phantom measurements using “raw SNR” (Equation (4)) of absolute inorganic phosphate (Pi) metabolite signal. **(A)**With approximately matched acquisition times, 3D UTE rosette’s mean SNR was 69% higher. **(B)** As both sequences share the same nominal resolution, example axial SNR maps show clear signal intensity across a width of 5 voxels (equivalent to 100 mm). **(C)** A uniform 100-mm diameter Pi bottle phantom was prepared and used for both measurements.

Figure 5 showcases results and setup of phantom experiments with dual-tuned flexible surface coil. With approximately matched acquisition times, mean raw SNR (Eq. (4)) was 69% higher in 3D UTE rosette than in weighted CSI.

### 3.3 *In vivo* leg comparison of UTE Rosette with Weighted CSI

**Figure 6:**
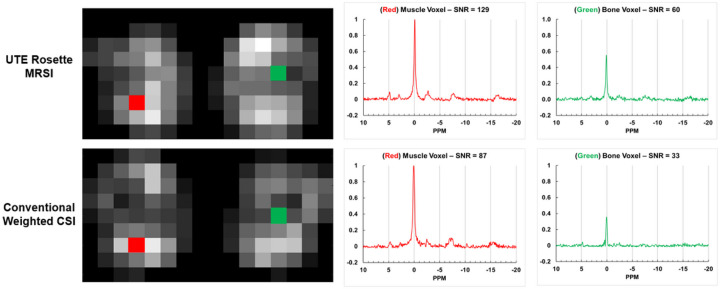
**(Left)** Example axial slice raw PCr SNR maps from both acquisitions for one quadriceps subject. Both protocols clearly discriminate between high signal muscle tissue and low signal femur region. **(Right)** Unfiltered magnitude spectra from each method in the highlighted muscle **(red)** and bone **(green)** voxels scaled to the maximum PCr peak amplitude. Stated spectral SNR is for PCr peak.

Figure 6 shows representative 3D UTE rosette and weighted CSI axial PCr maps and spectra in the same volunteer. High-signal muscle regions are clearly distinguishable from low-signal bony femur regions. As expected, PCr predominates the ^31^P muscle spectrum alongside smaller Pi and ATP peaks.

**Figure 7:**
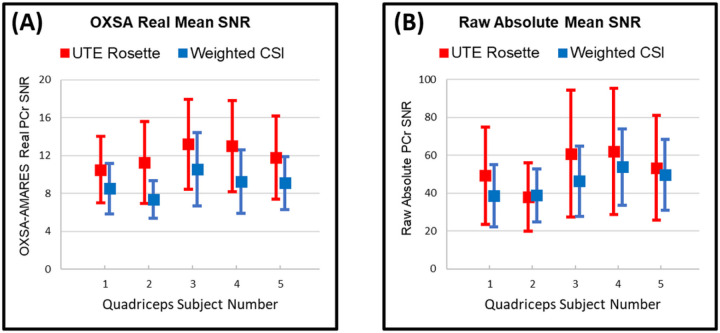
**(A)** Measured PCr SNR (mean ± SD) from OXSA-AMARES quantifiable voxels across all five quadriceps subjects using each acquisition scheme and real data (Equation (3)). By this quantification metric, 3D UTE rosette outperforms weighted CSI by approximately 34% *in vivo*. **(B)** Measured raw PCr SNR (mean ± SD) from quantifiable voxels across all five quadriceps subjects and bottle phantom using each acquisition scheme and absolute data (Equation (4)). By this quantification metric, 3D UTE rosette outperformed weighted CSI by approximately 69% in phantom and 18% *in vivo*. Detailed results are provided in Tables 2 and 3.

Quantitative PCr results for all subjects are given in Fig. 7, highlighting the different SNRs resulting from Equations (3) and (4) respectively. Tables 2 and 3 summarize these distinctions. While 3D UTE rosette consistently outperformed weighted CSI, the advantage was slightly more prominent in AMARES-fitting of the real data at 34% compared to raw SNR of absolute data at 18%.

## 4. Discussion

### 4.1 Experimental overview

This study demonstrates the feasibility of using 3D UTE ^31^P-MRSI with a novel rosette k-space trajectory to acquire quality *in vivo* human subject data. Simulations showed the rosette trajectory produced acceptable image quality and SRF characteristics when compared to a conventional 3D weighted CSI acquisition. Experimental phantom scans utilized a uniform Pi bottle solution and the same scanning parameters later applied to *in vivo* quadriceps subjects.

### 4.2 UTE advantages

The novel acquisition’s 70 μs acquisition delay is substantially lower than the 300–500 μs delays in previously published UTE ^31^P CSI methods^40,41^, minimizing transverse signal decay and first-order dephasing. Accurate phasing is key to spectral fitting and quantification of real spectral data; when fitting parameters must be tailored to hundreds of voxels across a large volume, such as for high resolution 3D MRSI, the challenge of avoiding phasing errors is most apparent. As expected, all 3D UTE rosette data were intrinsically devoid of noticeable first-order phasing, thereby streamlining the quantification process. The SNR gap between 3D UTE rosette and weighted CSI acquisitions was narrowed when solely considering absolute data (Eq. (4)). This distinction might be partially explained by the absence of phase in these magnitude spectra, whereby the 3D UTE rosette (T_E_ = 70 μs) acquisition loses a portion of its advantage over conventional weighted CSI (T_E_ = 2.3 ms). Notably, SDs for 3D UTE rosette SNR were significantly higher (50% or more) compared to weighted CSI. This elevated variation is partially explained by the novel acquisition’s significantly higher SNR; moreover, weighted CSI’s wider SRF engenders higher inter-voxel crosstalk, diminishing overall variation among quantified voxels.

### 4.3 Acceleration potential

Although these 36-minute acquisitions are quite lengthy, conventional ungated *in vivo* 3D ^31^P-MRSI typically requires a minimum of 20 minutes at 3T. Compared to a weighted Cartesian trajectory, this novel rosette k-space pattern’s relative incoherence makes it a very suitable candidate to CS acceleration via undersampling. Applying undersampling factors of 2 to 4, as demonstrated previously in uT_2_ brain imaging^25^, could reduce ^31^P-MRSI’s TA to 9–18 minutes (or less with fewer averages).^46^ Such an acceleration would allow implementation of ^31^P-MRS within realistic clinical constraints, while also being translatable to UHF research systems and higher resolutions.

As with all ^31^P-MRS, spectral quality can also see potential improvement via proton decoupling and nuclear Overhauser effect (NOE) enhancement, albeit with implications for SAR and measured metabolite ratios. Furthermore, appropriately applied low-rank approximation and principal component analysis denoising have seen use in heightening SNR of MRSI data sets;^47–49^ nevertheless, in the absence of ground truths or precise simulation, care must be taken in estimating metabolite concentration uncertainties after denoising.

### 4.4 Resolution and SBW

Many non-Cartesian acquisitions face restrictions in spatial resolution, SBW, and SNR due to available gradient hardware.^31^ For example, spiral trajectories face reduced SNR while waiting to return to k-space center between spirals; this inefficiency is addressed by closed-loop, out-in trajectories, but these remain impractical outside UHF animal gradient systems.^50^ Concentric rings can be similarly adjusted to meet needs with temporal interleaves.^36^

Still, SBW limitations remain a significant challenge; while 2.0 kHz might be sufficient for ^31^P-MRS at 3T, such a SBW would only offer a spectral range of around 17 ppm at 7T. While this rosette acquisition sampled 48 points per petal every 480 μs, the sequence remains highly customizable. By leveraging the second half of each petal (Fig. 1), it is possible to partially satisfy Nyquist criterion at even higher bandwidths and enable finer resolution reconstructions than the relatively coarse 8 mL voxels shown here. Additionally, this permits greater SBW acquisitions, opening the door to 3D UTE rosette ^31^P-MRSI at UHF and ^1^H MRSI at 3T. However, these petal halves are analogous to odd and even echoes of EPSI MRSI; since the timings between individual N_pp_ are not equidistant, “full-petal” spectra will suffer from some degree of noise amplification and aliasing artifact.

### 4.5 Other limitations

Further experimentation is required in exploring the potential and limitations of 3D UTE rosette MRSI. Notably, these quadriceps scans focused on quadriceps muscle with plentiful PCr signal in a healthy volunteer population. However, ^31^P-MRSI is frequently applied in measuring diverse brain, cardiac, and liver spectra, where nearby tissues may introduce contaminating metabolite signals. Minimal signal contamination was observed in noisy voxels within bony regions. Nonetheless, due to the relative uniformity of skeletal muscle spectra, it would be difficult to discern the rosette acquisition’s relatively incoherent aliasing. Future work will aim to assess accelerated performance in patient populations.

## 5. Conclusions

Using the quadriceps of five healthy volunteers at 3T, we investigated a potential application to ^31^P-MRSI using a novel 3D UTE rosette sequence. In comparison to a conventional 3D weighted CSI with matched bandwidth, nominal resolution, and acquisition time, the novel rosette acquisition provided competitive resolution and superior SNR with straightforward quantification. As this proof-of-concept study was limited to five subjects and a relatively homogeneous region of PCr-plentiful muscle, additional testing is required to demonstrate efficacy in differentiating diverse and diseased tissue regions.

## Data Availability

Data are available from the authors upon reasonable request.

## Figures and Tables

**Table 1:** Table of protocol parameters for conventional 3D weighted CSI, novel 3D UTE rosette MRSI, and additional retrospectively accelerated rosette sequences. Nominal voxel size was matched between methods, with total acquisition time approximately equal between the two full acquisitions. SBWs were matched via interpolation during post-processing.

SEQUENCE:	UTE Rosette	Weighted CSI
**T**_**A**_ **(mm:ss)**	36:00	36:56
**T**_**R**_	350 ms	1000 ms
**T**_**E**_	70 μm	2.3 ms
**Number of Averages**	4	4
**Reconstruction Matrix**	24×24×24	16×16×16
**Nominal Voxel (mL)**	8	8
**FOV (mm** ^**3**^ **)**	480×480×480	320×320×320
**Bandwidth (Hz)**	2083	2200
**Time Samples**	512	512
**T**_**A**_ **relative to CSI**	0.97	1.00

**Table 2:** Mean *in vivo* PCr SNR from quantifiable voxels in individual subjects using each method. With matched voxel size and acquisition resolution, 3D UTE rosette consistently outperforms weighted CSI acquisition in measured SNR.

OXSA-AMARES (Eq. 3)	UTE Rosette	Weighted CSI	Raw Absolute (Eq. 4)	UTE Rosette	Weighted CSI
Subject 1	10.50 (± 3.49)	8.51 (± 2.66)	Subject 1	49.19 (± 25.72)	38.50 (± 16.44)
Subject 2	11.26 (± 4.32)	7.37 (± 1.97)	Subject 2	37.96 (± 17.97)	38.77 (± 14.02)
Subject 3	13.21 (± 4.75)	10.54 (± 3.88)	Subject 3	60.74 (± 33.49)	46.31 (± 18.49)
Subject 4	12.99 (± 4.83)	9.24 (± 3.35)	Subject 4	61.96 (± 33.23)	53.75 (± 20.08)
Subject 5	11.78 (± 4.42)	9.11 (± 2.79)	Subject 5	53.35 (± 27.57)	49.62 (± 18.81)
Total Fitted Voxels	1134	947	Total Fitted Voxels	1325	1378
**Overall Leg PCr SNR**	**11.95** (± 4.39)	**8.95** (± 3.00)	**Overall Leg PCr SNR**	**52.83** (± 28.18)	**44.95** (± 17.70)

**Table 3:** Mean PCr SNR from quantifiable voxels across all five quadriceps subjects and bottle phantom using each method. With matched voxel size and acquisition resolution, 3D UTE rosette consistently outperforms weighted CSI acquisition in measured SNR.

SEQUENCE:	UTE Rosette	Weighted CSI	UTE/CSI SNR Ratio
OXSA-AMARES Real *in vivo* PCr SNR	**11.95** (±4.39)	**8.95** (± 3.00)	**1.34**
Raw Absolute *in vivo* PCr SNR	**52.83** (±28.19)	**44.95** (± 17.70)	**1.18**
Raw Absolute Phantom SNR	**52.48** (± 34.6)	**31.00** (± 9.84)	**1.69**
